# The Expression Levels of *TREX1* and IFN-α Are Associated with Immune Reconstitution in HIV-1-Infected Individuals

**DOI:** 10.3390/v16040499

**Published:** 2024-03-25

**Authors:** Maria Alice Freitas Queiroz, Allysson Quintino Tenório de Oliveira, Tuane Carolina Ferreira Moura, Wandrey Roberto dos Santos Brito, Emmanuelle Giuliana Mendes Santana, Lorena Leticia Peixoto de Lima, Felipe Teixeira Lopes, Carlos David Araújo Bichara, Ednelza da Silva Graça Amoras, Ricardo Ishak, Izaura Maria Vieira Cayres Vallinoto, Antonio Carlos Rosário Vallinoto

**Affiliations:** 1 Laboratory of Virology, Institute of Biological Sciences, Federal University of Pará, Belém 66.075-110, PA, Brazil; allyssonquintino@yahoo.com.br (A.Q.T.d.O.); tuanecfmoura@gmail.com (T.C.F.M.); wandrey.ben1@gmail.com (W.R.d.S.B.); emmanuelle.santana@icb.ufpa.br (E.G.M.S.); lorenalplima@gmail.com (L.L.P.d.L.); felip_lopes1@hotmail.com (F.T.L.); bichara@amaralcosta.com.br (C.D.A.B.); ednelza@hotmail.com (E.d.S.G.A.); rishak@ufpa.br (R.I.); ivallinoto@ufpa.br (I.M.V.C.V.); vallinoto@ufpa.br (A.C.R.V.); 2 Graduate Program in Biology of Infectious and Parasitic Agents, Federal University of Pará, Belém 66.075-110, PA, Brazil

**Keywords:** HIV-1, *TREX1*, polymorphisms, gene expression, IFN-alpha

## Abstract

TREX1 acts in the initial prevention of an autoimmune response, but it may contribute to the permissiveness of retrovirus infections. This study investigated the association between the levels of *TREX1* gene expression with the polymorphisms *TREX1* rs3135941 (T/C) and *TREX1* rs3135945 (G/A), and the presence of antinuclear antibodies (ANA) in antiretroviral therapy (ART)-naïve individuals and after 1 year of treatment. Blood samples from 119 individuals with HIV-1 were subjected to genotyping of polymorphisms and quantification of *TREX1* gene expression and HIV-1 viral load by qPCR. The concentration of IFN-α and the number of CD4^+^/CD8^+^ T lymphocytes were determined by ELISA and flow cytometry, respectively; ANA was investigated by immunofluorescence. A control group of 167 seronegative individuals was used for the comparison of genotypic frequencies. The frequency of the polymorphisms were not associated with HIV infection or with variations in the expression of *TREX1* and IFN-α (*p* > 0.05). ART-naïve individuals exhibited higher TREX1 expression and lower IFN-α expression. After 1 year of ART, *TREX1* levels were reduced, while IFN-α and CD4^+^ T lymphocytes were elevated (*p* < 0.05). Some individuals on ART presented ANA. These results suggest that ART-mediated restoration of immune competence is associated with a reduction in *TREX1* expression, which may induce the development of ANA, regardless of the polymorphism investigated.

## 1. Introduction

Despite all the advances in the control of HIV-1 infection, the virus remains a major public health problem worldwide, with an estimated 1.3 million new infections each year [1]. Because there is still no treatment available to eradicate the infection, the main control measure is antiretroviral therapy (ART), which promotes a reduction in viral load to undetectable levels, allowing the restoration of the individual’s immune response [2,3]. Unfortunately, ART may contribute to the development of metabolic changes and autoimmune reactions [3,4,5,6].

The development of autoimmune diseases is mainly attributed to genetic and environmental factors that are responsible for triggering the dysfunction of the immune response [7]. Among the environmental factors, viruses are primarily responsible for triggering autoimmune events in genetically susceptible individuals. The mechanisms related to the breakdown of self-tolerance include molecular mimicry and the activation of a marked and nonspecific antiviral immune response, which can induce the formation of self-reactive B and T cells [8]. In addition to the HIV-1 infection itself being able to induce a drop in self-tolerance, treatment with antiretrovirals (ARVs) has been associated with the development of autoimmune manifestations by promoting the reestablishment of an individual’s immunological competence [9].

Some cytosolic sensors involved in innate immunity may act in the initial prevention of an autoimmune response [10,11]. In infections, the activity of these sensors may contribute to the restriction or permissiveness of retroviral infections [12,13,14]. The three-prime repair exonuclease 1 (TREX1) protein is a sensor that promotes the degradation of DNA dispersed in the cytoplasm (ssDNA and dsDNA), both of endogenous and exogenous origin, contributing to the prevention of autoimmune diseases [12]. TREX1 activity may favor the persistence of some viral infections and is considered a factor of retroviral permissiveness [13,15]. The TREX1 activity inhibits the recognition of viral DNA by the cyclic GMP-AMP synthase (cGAS) sensor and the activation of the cGAS-STING pathway, resulting in reduced expression of IFN-I, which is essential for the control of viral infections [16,17].

Genetic variations in different cytosolic sensors that may influence the course of autoimmune diseases and viral infections have been investigated [18,19]. Polymorphisms in the *TREX1* gene were associated with autoimmune manifestations, such as systemic lupus erythematosus and Sjögren’s syndrome [20,21], and with retrovirus infection [6,18,22]. Among the various polymorphisms located in the *TREX1* gene, some stand out in HIV-1 infection; the *TREX1* rs3135941 (T/C) polymorphism was related to faster progression of the infection [23], and the *TREX1* rs3135945 (G/A) polymorphism was associated with susceptibility to virus infection [22]. The *TREX1* rs11797 (C/T) variation is associated with the maintenance of the immune status of individuals with HIV-1 [6]. However, in human T-cell leukemia virus type 1 (HTLV-1) infection, the *TREX1* rs11797 (C/T) polymorphism was associated with a high proviral load [18].

Although different *TREX1* gene polymorphisms have been associated with retroviral infections, neither the relationship between *TREX1* gene expression level and its polymorphisms nor the influence of *TREX1* gene expression levels on the development of antinuclear antibodies (ANAs) in HIV-1 infection have been determined. This study investigated the association of variations in *TREX1* gene expression levels with the *TREX1* rs3135941 (T/C) and *TREX1* rs3135945 (G/A) polymorphisms and the presence of ANA in ART-naïve individuals and after 1 year of treatment.

## 2. Materials and Methods

### 2.1. Type of Study, Population, and Sample Collection

In this longitudinal study, we evaluated a cohort of 119 ART-naïve people living with HIV-1 who were followed up and evaluated twice, namely before starting therapy and after 1 year of ART use. The individuals were from the outpatient clinic of the Casa Dia Testing and Counseling Center (CAT/SAE), located in the city of Belém, Pará, Brazil. The inclusion criteria for individuals in the cohort were age ≥ 18 years, being of either sex, and not having used antiretroviral drugs. The exclusion criteria were autoimmune disease, coinfection with hepatitis B virus, hepatitis C virus, hepatitis D virus or HTLV-1/2, use of corticosteroids, and clinical diagnosis of AIDS. Individuals who did not undergo treatment for 1 year of the study were also excluded.

To compare the genotypic and allelic frequencies of polymorphisms in the *TREX1* gene, a group of 167 blood donors seronegative for HIV, HBV, HCV, HTLV, Chagas disease, and syphilis was used. This group was called the control group, and individuals were selected by matching for age and sex with the individuals in the HIV-1 group.

Blood samples were collected in two tubes (5 mL each) containing ethylenediaminetetraacetic acid (EDTA) as an anticoagulant. The samples were transported to the Laboratory of Virology of the Federal University of Pará, where they were subjected to aliquot separation of whole blood, leukocytes, and plasma. Whole blood samples were used for quantification of CD4^+^ and CD8^+^ T and genomic DNA extraction. Plasma samples were used to quantify the HIV-1 plasma viral load and IFN-α concentration. Leukocyte samples were subjected to RNA extraction.

### 2.2. Ethics Approval

The project was approved by the Ethics Committee of the Oncology Research Center of the Federal University of Pará (CAAE 66529116.4.0000.5634). All the subjects were informed about the project. All participants were adults, and those who agreed to participate had to sign an informed consent form.

### 2.3. DNA Extraction

Genomic DNA was extracted from peripheral blood leukocytes using the Puregene™ Kit (Gentra Systems, Minneapolis, MN, USA) following the steps of cell lysis, protein precipitation, DNA precipitation, and DNA hydration. All processing steps followed the manufacturer’s recommendations.

### 2.4. Genotyping of TREX1 rs3135941 (T/C) and TREX1 rs3135945 (G/A)

Genotyping was performed by real-time PCR using the StepOnePLUS™ Real-Time PCR System (Thermo Fisher, Carlsbad, CA, USA). The reaction was performed using the TaqMan™ *TREX1* assay 3135941 (C__32390138_10) and rs3135945 (C__32390141_10), obtained commercially and containing specific primers and probes for amplification of the target sequence (Thermo Fisher, Carlsbad, CA, USA). The reaction consisted of 1× MasterMix, H_2_O, 20× assay, and 50 ng of DNA. The cycling program was 10 min at 95 °C, 40 cycles of 15 s at 95 °C, and 1 min at 60 °C.

### 2.5. RNA Extraction and Reverse Transcription

Total RNA was extracted from peripheral blood leukocytes using the total RNA extraction kit TRIzol™ Plus RNA Purification Kit (Thermo Fisher Scientific, Waltham, MA, USA). The concentration of the extracted RNA was determined using a BioDrop™ (Bio-Rad, Hercules, CA, USA) according to the manufacturer’s instructions. The concentrations of total RNA were set to 50 ng/µL for the synthesis of complementary DNA (cDNA).

The extracted RNA was converted into cDNA using the High-capacity cDNA Reverse Transcription^®^ with RNase Inhibitor kit (Applied Biosystems, Foster City, CA, USA). For the cDNA reaction, a mixture with a final volume of 20 µL was prepared containing 2 µL of 10× RT Buffer, 0.8 µL of 25× dNTP Mix (100 nM), 2 µL of random primer, 1 µL of MultiScribeTM Reverse Transcriptase, 1 µL of RNase OnceTTM, 3.2 µL of ultrapure water supplied by the kit, and 10 µL of extracted RNA. The mixture was subjected to PCR in a Mastercycler Personal thermal cycler (Eppendorf, Hamburg, Germany) with cycles of 25 °C for 10 min, 37 °C for 120 min, and 85 °C for 5 min.

### 2.6. Quantification of Gene Expression

*TREX1* gene expression was performed by quantifying the mRNA using the real-time PCR (qPCR) technique. Initially, the standardization of the qPCR with the cDNA and probes (endogenous and target genes) was performed to calculate the efficiency of the amplification reactions. In the standardization reactions, different concentrations of cDNA were tested (pure and in 4 dilutions of factor 2, 1:2, 1:4, 1:8, and 1:16). All the reactions were performed on plates and in triplicate, and the same cDNA (at different dilutions) was analyzed with the different probes to draw an efficiency curve to validate the 2-ΔΔCT analytical method (∆∆Ct = ∆Csample − ∆Ctreference). All tests showed the expected efficiency (100% ± 10) [24].

The relative quantification of gene expression consisted of the amplification of the target gene with the endogenous gene (normalizer) using TaqMan™ assays (Applied Biosystems, Foster City, CA, USA) and the StepOnePLUS™ Real-Time PCR System (Thermo Fisher Scientific, Waltham, MA, USA). The reactions were performed in a single-plex format according to the manufacturer’s protocol. The test used for *TREX1* expression was Hs03989617, and the glyceraldehyde-3-phosphate dehydrogenase (*GAPDH*) gene (Hs02786624_g1) was used as an endogenous control. All the products were obtained commercially (Thermo Fisher Scientific, Waltham, MA, USA). For the reaction, 15 µL of 2× TaqMan^®^ Universal PCR Master Mix, 1.5 µL of 20× TaqMan Gene Expression Assays, 3 µL of cDNA and 10.5 µL of RNase-free water were used. The thermocycling program was 2 min at 50 °C, 10 min at 95 °C, and 1 min at 60 °C. The relative quantification (RQ) of the expression of target genes was determined using the comparative CT method (∆∆Ct), using the formula 2−ΔΔCT, where ∆∆Ct = ∆Ct sample − ∆Ct reference (Life Technologies, Carlsbad, CA, USA).

### 2.7. Quantification of CD4^+^ and CD8^+^ T Lymphocytes and the HIV-1 Plasma Viral Load

The CD4^+^ and CD8^+^ T lymphocyte counts were performed at the Virology Laboratory, as established by the National Network of CD4^+^/CD8^+^ T lymphocytes of the Ministry of Health. The flow cytometry methodology was used, which evaluated a whole blood sample with EDTA, using BD FACScount equipment and BD multi-test reagents (CD45, CD3, CD4, and CD8) (BD, Franklin Lakes, NJ, USA), following the protocol recommended by the manufacturer.

The plasma viral load was determined at the Virology Laboratory, following the standard methodology of the National Viral Load Network of the Ministry of Health, based on qPCR technology, using the Sample Purific CV HIV-1 Extraction Kit, the HIV-1 Viral Load Amplification, and the Abbott 2000mrt thermal cycler (ABBOTT, Chicago, IL, USA) and all steps were followed according to the manufacturer’s recommendations. Viral load measurements were used in log10.

### 2.8. Plasma Concentration of IFN-α

IFN-α levelswere quantified by enzyme-linked immunosorbent assay (ELISA) using the IFN alpha Human ELISA Kit (Thermo Fisher, Waltham, MA, USA), which uses specific monoclonal antibodies to detect cytokines. The test was performed according to the manufacturer’s recommendations.

### 2.9. ANA Research in HEp-2 Cells

The plasma samples were tested using the commercial VIRGO^®^ ANA/HEp-2 IgG indirect fluorescent antibody (IFA) kit (Hemagen Diagnostics, Columbia, MD, USA) following the manufacturer’s specifications. The slides were read under an Eclipse E-200 immunofluorescence microscope (Nikon, Minato, Tokyo, Japan) using a 10× ocular lens and a 40× objective lens. To record and store the images obtained from the fluorescence standards, a ZOE Fluorescent Cell Imager was used (Bio-Rad, Hercules, CA, USA).

### 2.10. Statistical Analysis

The obtained information was entered into a database in the Microsoft Office Excelsoftware version 2019. The allelic and genotypic frequencies of the polymorphisms were determined by direct counting, and the differences between the HIV-positive and control groups were assessed using the chi-square test or G test. The Hardy–Weinberg equilibrium test was performed to assess the expected frequency of *TREX1* rs3135941 (T/C) and *TREX1* rs3135945 (G/A) genotypes in each group. The normality of quantitative data was assessed using the Shapiro–Wilk test. Continuous variables before and after 1 year of ART use were compared using the Wilcoxon test, and comparison among the genotypes for *TREX1* rs3135941(C/T) was performed using the Kruskal–Wallis test. Viral load was compared using the chi-squared test or G test. All tests were performed in Biostat 5.3 software version 5.3 or GraphPad Prism 8.0. Statistical tests with *p* values < 0.05 were considered significant.

## 3. Results

The mean age of individuals with HIV-1 was 39.17 years and the majority were male (61.54%). A comparison of the genotypic and allelic frequencies of *TREX1* rs3135941 (T/C) and *TREX1* rs3135945 (G/A) between the HIV-1 and control groups showed that the HIV-1 group had a higher frequency of the polymorphic genotypes (CC and CT) for the *TREX1* rs3135941 (T/C) genetic variation, but these differences were not statistically significant. For *TREX1* rs3135945 (G/A), the genotype distribution was very similar between the two groups (Table 1).

**Figure 1:**
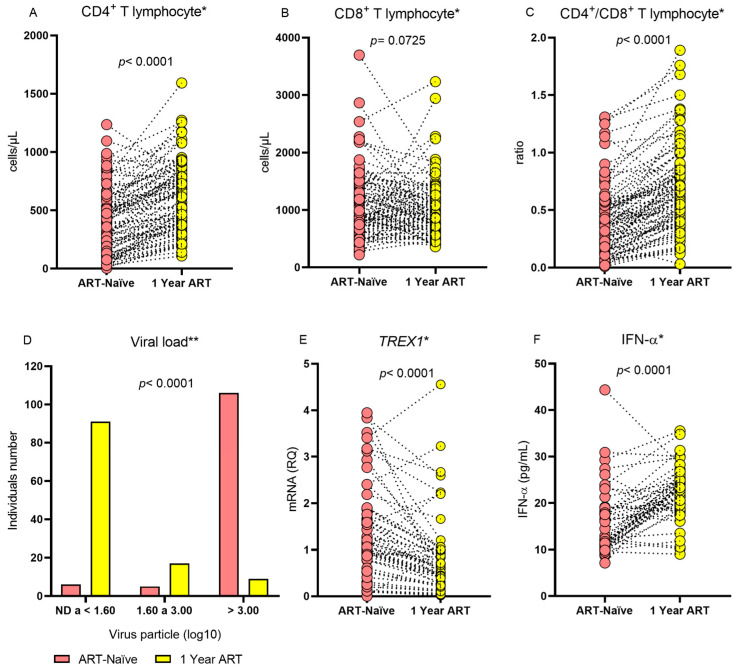
Comparison of the levels of (**A**) CD4^+^ T lymphocytes, (**B**) CD8+ T lymphocytes, (**C**) CD4^+^/CD8^+^ T lymphocyte ratio, (**D**) frequency of viral load levels, (**E**) TREX1 gene expression levels, and (**F**) IFN-α concentration. * Wilcoxon test; ** chi-squared test.

The evaluation of individuals with HIV-1 before and after 1 year of treatment showed that the median CD4^+^ T lymphocyte count and the CD4^+^/CD8^+^ T lymphocyte ratio were higher during ART use and that individuals were more likely to have a lower viral load during the same period. After 1 year of therapy, the median *TREX1* gene expression was lower and the IFN-α level was higher (Table 2 and Figure 1). The therapeutic regimens applied to the patients were tenofovir/lamivudine/efavirenz (n = 117; 98.32%), atazanavir/tenofovir/lamivudine/ritonavir (n = 1; 0.84%), and tenofovir/lamivudine/dolutegravir (n = 1; 0.84%).

**Figure 2:**
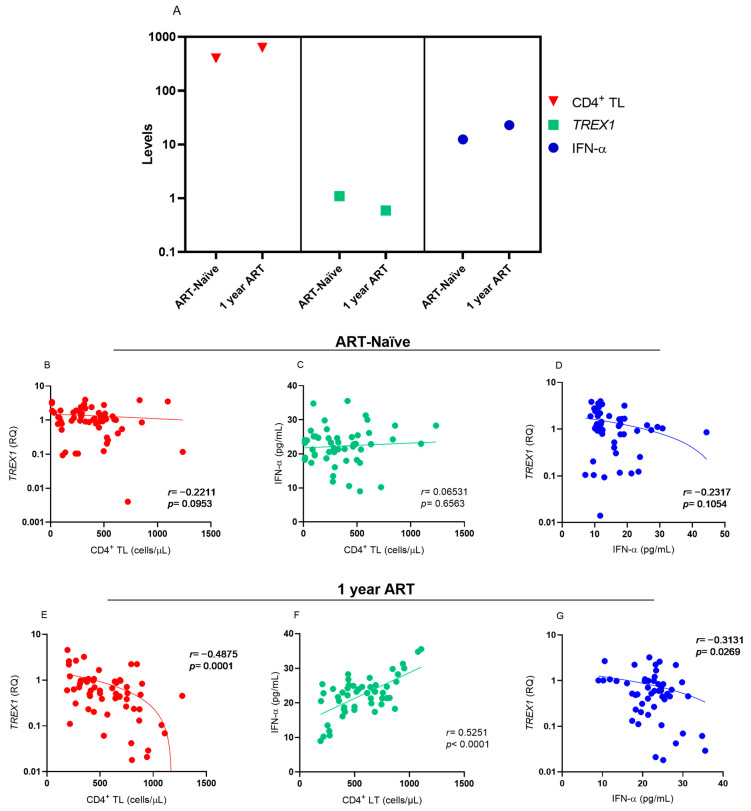
(**A**) Median levels of CD4^+^ T lymphocytes, *TREX1*, and IFN-α in individuals infected with HIV-1 before and after 1 year of ART. Correlation between the levels of (**B**) CD4^+^ T lymphocytes and TREX1, (**C**) CD4^+^ T lymphocytes and IFN-α and (**D**) IFN-α and *TREX1* before ART and (**E**–**G**) after 1 year of therapy. Spearman test.

Analysis of the levels of CD4^+^ T lymphocytes, *TREX1* and IFN-α showed that the increase in CD4^+^ T lymphocyte levels was related to the reduction in *TREX1* levels and an increase in IFN-α levels after one year of ART use (Figure 2A). Before starting ART, no correlation was observed between the markers evaluated (Figure 2B–D). During the one-year period of ART, there was a negative correlation between the levels of CD4^+^ T lymphocytes and *TREX1* expression (Figure 2B), a positive correlation between the levels of CD4^+^ T lymphocytes and IFN-α (Figure 2C), and a low negative correlation between *TREX1* and IFN-α expression levels (Figure 2D).

**Figure 3:**
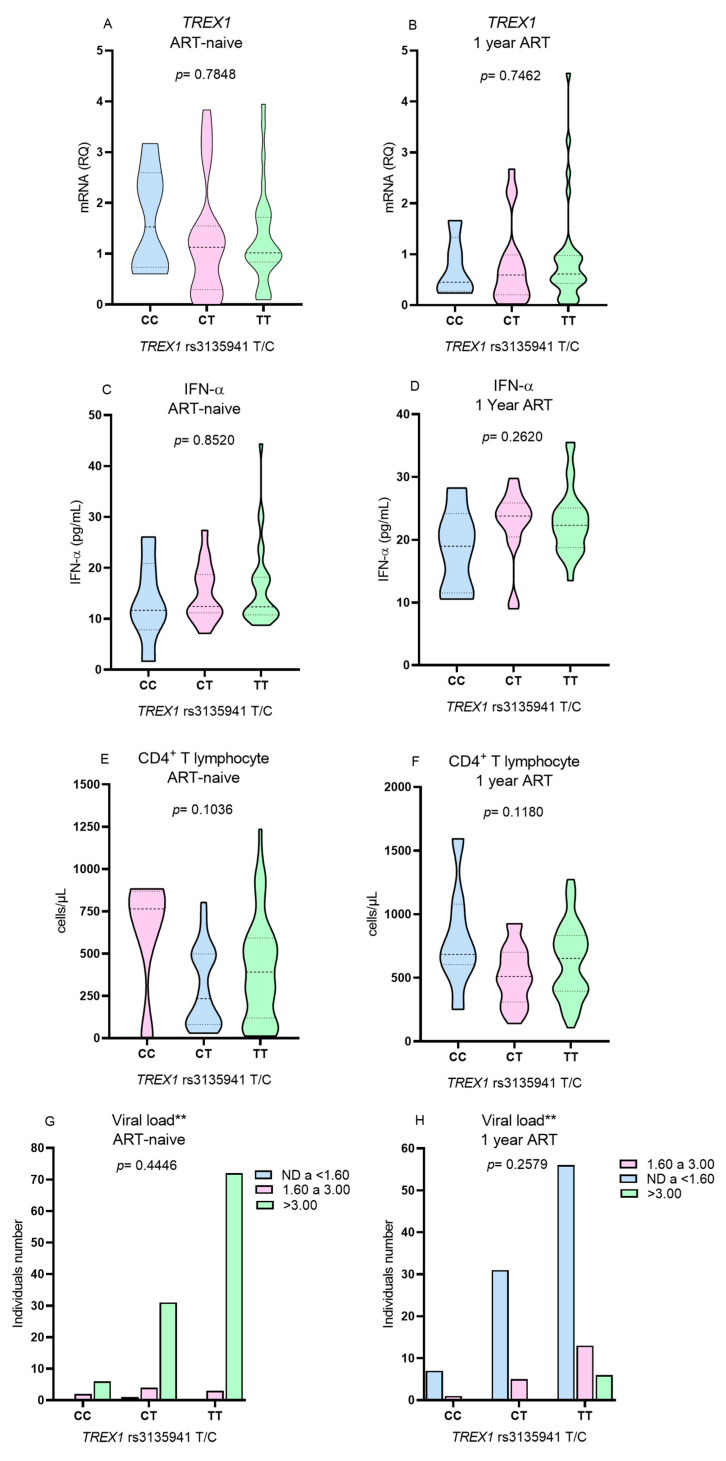
Comparison of the levels of TREX1 gene expression, IFN-α, CD4^+^ T lymphocytes, and viral load according to the genotype for the TREX1 rs3135941 T/C polymorphism (**A**,**C**,**E**,**G**) before the use of ART and (**B**,**D**,**F**,**H**) after 1 year of therapy. ** G test.

Due to the low frequency of the polymorphic genotype for the *TREX1* rs3135945 (G/A), the T lymphocyte count, viral load, *TREX1* expression and IFN-α levels were evaluated only in relation to the genotypes for the *TREX1* rs3135941 polymorphism (T/C). No differences were observed in the levels of the investigated variables among individuals with different genotypes of *TREX1* rs3135941 (T/C) before the initiation of ART (Figure 3A,C,E,G) or after 1 year of therapy (Figure 3B,D,F,H).

**Figure 4:**
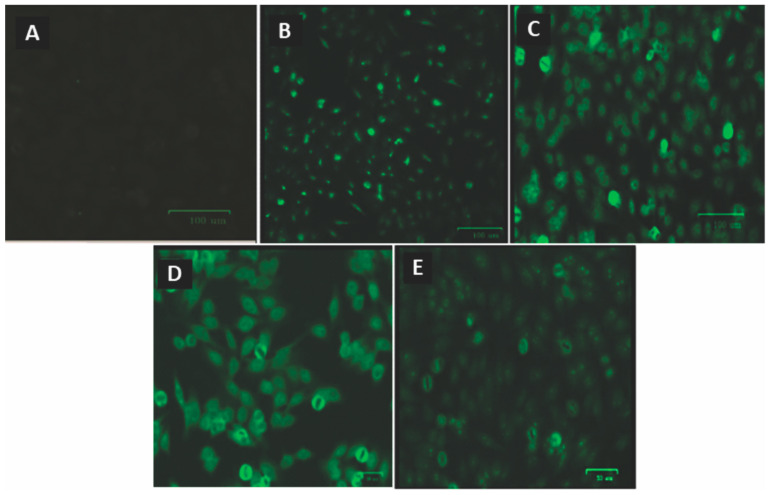
Fluorescence patterns of antinuclear antibodies in ANA^+^ HEp-2 cells from HIV-1 individuals after the use of ART. (**A**) Negative control, (**B**) fine dotted nuclear, (**C**) dotted nucleolar, (**D**) cytoplasmic, and (**E**) nucleolar with isolated dots.

Of all the samples tested for ANA, none showed positive fluorescence before ART. After using ART, seven individuals exhibited different fluorescence patterns (Figure 4).

**Figure 5:**
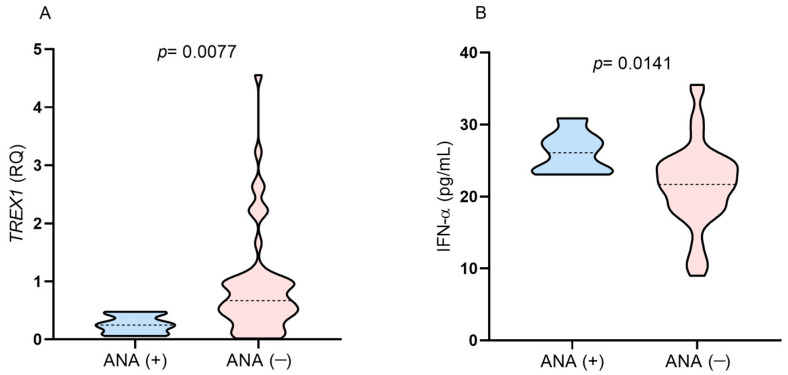
Comparison of the levels of (**A**) *TREX1* gene expression and (**B**) IFN-α between individuals with positive and negative results for ANA after 1 year of ART.

Table 3 shows the ANA fluorescence pattern of each individual, the genotypes of the polymorphisms investigated, and the *TREX1* and IFN-α expression levels. Among the seven individuals with ANA, two had the TT genotype, two had the CC genotype, three had the CT genotype for the *TREX1* rs3135941 (T/C), and all were homozygous for the wild-type genotype (GG) for the *TREX1* rs3135945 (G/A). ANA-positive individuals had *TREX1* gene expression levels below the median in the studied group and higher plasma IFN-α levels (Figure 5A,B). All the subjects were on a regimen containing tenofovir/lamivudine/efavirenz.

**Table 3:** Characterization of patients with positive ANA according to the genotypes of the polymorphisms investigated and the levels of *TREX1* and IFN-α.

Patient	Fluorescence Standards	*TREX1* rs3135941/rs3135945 Genotypes	*TREX1* Gene Expression (RQ)	IFN-α (pg/mL)
#1	Fine dotted nuclear	TT/GG	0.10	30.87
#2	Fine dotted nuclear	CC/GG	0.30	27.38
#3	Nucleolar with isolated dots	CT/GG	0.44	23.48
#4	Dotted nucleolar	TT/GG	0.20	23.91
#5	Fine dotted nuclear	CT/GG	0.25	23.04
#6	Dotted nucleolar	CC/GG	0.06	26.09
#7	Cytoplasmic	CT/GG	0.48	28.26

## 4. Discussion

Autoimmune manifestations may result from negative dysregulation of cytosolic sensors, such as in cGAS and STING, which leads to marked activation of IFN-I and NF-κB signaling. In this context, TREX1 acts by degrading cytosolic DNA, inhibiting the cGAS-STING pathway and preventing the development of an autoimmune response. On the other hand, in HIV infection, TREX1 activity promotes a decrease in viral DNA below the threshold of immune activation, inhibiting the adequate activation of cGAS-STING and the production of IFN-I, which are essential for the control of viral infections [25].

Our study evaluated *TREX1* gene polymorphisms, *TREX1* gene expression, plasma IFN-α levels, and the presence of ANA in the context of HIV-1 infection and ART use. The genotype and allele distributions of the *TREX1* rs3135941 (T/C) and *TREX1* rs3135945 (G/A) polymorphisms were not associated with HIV-1 infection, and there was no association between the polymorphisms and the *TREX1* gene expression, IFN-α, and CD4^+^ T lymphocytes levels. Thus, our results suggest that the polymorphisms do not influence the immune response to HIV infection in the investigated group. However, in contrast, previous studies showed an association between the *TREX1* rs3135941 polymorphism (T/C) and the speed of progression of HIV-1 infection as result of increased virus replication, indicating that the *TREX1* rs3135941 polymorphism (T/C) may promote higher exonuclease activity, which would result in reduced IFN-I production [23,26]. The *TREX1* rs3135945 polymorphism (G/A) has been associated with susceptibility to HIV-1 infection in individuals of Caucasian descent [22].

The differences in the results between our study and other studies that did find an association between polymorphisms, and HIV-1 infection may be related to the ethnicity of the groups evaluated in each study. The *TREX1* rs3135941 (T/C) polymorphism was evaluated in individuals from Amsterdam and Iran [23,26], while the *TREX1* rs3135945 (G/A) polymorphism was evaluated in individuals from Italy [22]. The group investigated in the present study originates from the city of Belém, which is part of the Brazilian Amazon. This is a mixed-race population with a genetic contribution from white Europeans, black Africans, and native indigenous peoples [27]. Thus, it is possible that the frequencies of the investigated polymorphisms may be associated with HIV-1 infection only in certain ethnic groups. Another example is the *TREX1* polymorphism rs11797 (C/T), which was associated with HIV-1 infection in the same population as in the previous study [6], but not in the study that evaluated the polymorphism in a population of different ethnicities [22].

Differences in results among studies may also be related to the characterization of groups with HIV-1. We included individuals who used ART for 1 year; in the study by Booimam et al. (2014) and Tohidi et al. (2023), the majority of individuals were not using ART [23,26]. In this way, these two studies were able to evaluate the relationship of the *TREX1* rs3135941 (T/C) polymorphism with immunological and virological markers in natural HIV-1 infection over a period of time; our study evaluated the influence of polymorphisms in relation to markers by comparing periods of absence and presence of ART.

Due to the frequency of the polymorphic allele for *TREX1* rs3135941 (T/C) being slightly higher in the group with HIV-1, with a p value close to the significance value of the test, it is possible that the lack of association of the *TREX1* rs3135941 (T/C) polymorphism with HIV-1 infection may be related to our sample size. Furthermore, other factors may have contributed to determining the differences in results among the studies evaluated, such as gender, time of infection and, mainly, the use of ART.

In our study, no differences were observed in the levels of *TREX1* gene expression among individuals with different genotypes for the *TREX1* rs3135941 (T/C) polymorphism. Other authors evaluated the gene expression levels among genotypes for the *TREX1* rs3135941 (T/C) polymorphism in different conditions, including patients with Sjögren syndrome [21], HIV-1 [23], and healthy individuals [28], but they also found no differences in *TREX1* expression levels between genotypes. The *TREX1* rs3135941 (T/C) polymorphism was also not associated with plasma IFN-α levels in our study or in autoimmune diseases [21]. Taken together, these findings show that the *TREX1* rs3135941 (T/C) polymorphism located in the 5′ untranslated region (5′UTR) does not promote variations in *TREX1* expression levels, regardless of ethnic differences or the presence or absence of autoimmune or infectious diseases.

In contrast, the *TREX1* gene expression levels and plasma IFN-α concentrations of HIV-1 individuals who were ART-naïve were different from those after 1 year of therapy. In the period in which the individuals were ART-naïve, the expression of *TREX1* and the viral load were higher, while the number of CD4^+^ T lymphocytes, the CD4^+^/CD8^+^ T lymphocyte ratio, and the plasma IFN-α concentration were lower than when ART was used. These results reflect the activity of TREX1 in retroviral infection because in the absence of ART, there is more viral DNA, which induces greater expression of exonucleases and a reduction in IFN-α. Although TREX1 can degrade the HIV-1 DNA reverse transcript before it can integrate into the host cell genome, this activity is not sufficient to eliminate the infection, since TREX1 contributes to maintaining DNA levels undetectable by cGAS and STING sensors, leading to a reduction in IFN-I synthesis [12,25]. On the other hand, after 1 year of treatment with ART, the viral load decreased, leading to a reduction in the expression of *TREX1*. In this case, low levels of HIV-1 DNA appear to be enough to activate the cytosolic sensors responsible to produce IFN-I, including IFN-α. Furthermore, the increased levels of CD4^+^ T lymphocytes (median above 500 cells/µL) observed after the use of ART was associated with reduced levels of *TREX1* and increased levels of IFN-α, showing that immune reconstitution of infected individuals induced variations in the levels of these two markers, since before the use of ART, when individuals had levels of CD4^+^ T lymphocytes below 500 cells/µL, *TREX1* expression levels were higher and levels of IFN-α were lower. Thus, our findings contribute to a better understanding of the participation of TREX1 in natural HIV-1 infection and during ART intervention.

The presence of ANA was not related to the *TREX1* rs3135941 (T/C) or *TREX1* rs3135945 (G/A) polymorphisms since the individuals presented different genotypes for the *TREX1* rs3135941 (T/C) and only the wild-type genotype for *TREX1* rs3135945 (G/A). However, individuals with ANA had lower *TREX1* expression levels and higher IFN-α plasma levels than the median of the investigated group. Given that ANA were present in individuals after ART, who had higher IFN-α levels, these individuals may have experienced a more pronounced recovery of immune competence, which proves to be independent of the type of therapeutic regimen used. Other studies have described the association of ART use with the recovery of immune competence and the development of ANA [3,6]. Screening for ANA in individuals with HIV-1 infection who are receiving ART is important for identifying who could be at risk for developing possible autoimmune manifestations [6].

## 5. Conclusions

The *TREX1* rs3135941 (T/C) and *TREX1* rs3135945 (G/A) polymorphisms were not associated with susceptibility to HIV-1 infection, with variations in the levels of *TREX1* gene expression, IFN-α, or with the presence of ANA in the investigated population. *TREX1* gene expression was higher in ART-naïve HIV-1 individuals, suggesting marked TREX1 activity in natural HIV-1 infection. In contrast, after 1 year of ART, the reestablishment of immune competence characterized by reduced *TREX1* gene expression and high IFN-α and CD4^+^ T lymphocytes levels may be related to the development of ANA in some individuals.

## Figures and Tables

**Table 1:** Comparison of the genotypic and allelic frequencies for the *TREX1* rs3135941 (T/C) and *TREX1* rs3135945 (G/A) polymorphisms between HIV-1 individuals and control.

Genotypes and Alleles	HIV-1 n = 119 n (%)	Control n = 147 n (%)	*p*
*TREX1* rs3135941 (T/C)			
CC	8 (6.7)	5 (3.40)	0.1807 ^a^
CT	36 (30.25)	35 (23.81)	
TT	75 (63.03)	107 (72.79)	
* C	0.22	0.15	0.0672 ^a^
* T	0.78	0.85	
*TREX1* rs3135945 (G/A)			
AA	1 (0.84)	1 (0.68)	0.7890 ^b^
AG	10 (8.40)	9 (6.12)	
GG	108 (90.76)	137 (93.20)	
* A	0.05	0.04	0.6038 ^a^
* G	0.95	0.96	

n: number of individuals; * alleles; ^a^ chi-square test; ^b^ G test.

**Table 2:** Demographic and laboratory characteristics of HIV-1-infected individuals.

Variables	ART-Naïve	1 Year ART
**Age**, mean (SD)	39.17 (11.72)	-
**Sex**, n (%)		
Female	45 (38.46)	-
Male	72 (61.54)	
**CD4^+^ TL**, median (IQR)	399 (458)	626 (404)
**CD8^+^ TL**, median (IQR)	891 (746)	872 (515)
**CD4^+^/CD8^+^**, median (IQR)	0.35 (0.40)	0.65 (0.45)
**Viral load**—log_10_, n (%)		
ND to < 1.60	6 (5.12)	91 (77.78)
1.60 to 3.00	5 (4.28)	17 (14.53)
>3.00	106 (90.60)	9 (7.69)
***TREX1* expression**, median (IQR)	1.09 (1.06)	0.59 (0.72)
**IFN-α**, median (IQR)	12.37 (7.50)	22.87 (6.25)

## Data Availability

The data are available from the corresponding author upon reasonable request.
